# Effects of internet-based exercise intervention on depression and anxiety: A systematic review and meta-analysis

**DOI:** 10.1097/MD.0000000000037373

**Published:** 2024-02-23

**Authors:** Zuo Chen, Hui Huang, Ruidong Liu, Zhengyan Tang

**Affiliations:** a Division of Sports science and Physical Education, Tsinghua University, Beijing, China; b Division of Sociology, Tsinghua University, Beijing, China; c Sports Coaching College, Beijing Sport University, Beijing, China.

**Keywords:** anxiety, depression, internet-based exercise, mental health, physical activity

## Abstract

**Background:**

While exercise is acknowledged for its positive effects on depression and anxiety symptoms, the benefits of internet-based exercise on mental health have not been extensively examined. This study seeks to systematically review and quantify the outcomes of high-quality randomized controlled trials (RCTs) that investigate the impact of internet-based exercise on depression and anxiety symptoms.

**Methods:**

Following the PRISMA 2020 guidelines, we conducted a comprehensive meta-analysis of RCTs. Databases, including Web of Science Core Collection, PubMed, PsycINFO, Medline, BIOSIS Previews, SPORTDiscus, and Education Source, were scoured through in September 2023. After quality assessment and data extraction, the analysis was performed using R. Using random effects models, effect sizes were determined and subsequently represented as standardized mean differences (SMD).

**Results:**

Our analysis incorporated data from 11 RCTs, involving a cohort of 1009 participants. We observed a modest yet significant reduction in depression and anxiety symptoms, with an SMD of −0.44 [95% confidence interval (CI) (−0.63, −0.26), I^2 = 79.3%, *P* < .01]. Interestingly, the effects were more pronounced in individuals diagnosed with depression, as indicated by an SMD of −0.96 [95% CI (−1.55, −0.37), I^2 = 82%, *P* < .01]. Furthermore, participants utilizing smartphone applications as part of their intervention reported a meaningful reduction in their symptoms, evidenced by an SMD of −0.52 [95% CI (−0.90, −0.14), I^2 = 87%, *P* < .01]. Additionally, short-term interventions, specifically those lasting <12 weeks, indicated a notable alleviation in depression symptoms, with an SMD of −0.76 [95% CI (−1.38, −0.14), I^2 = 86%, *P* < .01].

**Conclusion:**

Internet-based exercise interventions yield significant amelioration in depression and anxiety symptoms, with heightened efficacy observed among individuals with depression. Notably, short-term interventions, specifically those under 12 weeks, demonstrate enhanced benefits for depression relief.

## 1. Introduction

The COVID-19 pandemic has extended its impact beyond the direct health implications of the virus. This situation has profoundly affected various determinants of mental health and physical activity, including social restrictions, fear of contagion, economic challenges, diminished outdoor activities, and shifts in governmental policies. These factors have significantly influenced mental health, particularly exacerbating conditions such as depression and anxiety.^[1]^

Mental health, an essential aspect of overall well-being, holds paramount importance for every individual.^[2]^ Depression and anxiety symptoms can severely diminish life quality, impacting familial dynamics.^[3]^ Notably, mental health disorders have been linked to grave consequences, such as the finding that over 90% of suicides in Western countries involve underlying mental health issues.^[4]^ The prevalence of these conditions poses challenges to societal stability and economic progress.^[5–7]^ For instance, a study conducted in China during the early stages of the COVID-19 pandemic reported that over half of the participants experienced considerable mental distress, with nearly 50% exhibiting moderate to severe anxiety or depression symptoms.^[8]^

The therapeutic potential of exercise for mental health has been a focal point of research for decades. Current evidence suggests that physical activity, particularly aerobic exercises,^[9]^ positively affect mental health.^[10]^ Investigations indicate that exercise as a non-pharmacological intervention provides significant benefits, especially to individuals with mental health disorders.^[11]^ Moreover, increasing physical activity levels correlates with reduced symptoms of depression and anxiety, supporting the use of exercise-based interventions for mental health improvement.^[12]^

Advances in technology, such as sophisticated website interfaces, smartphones, video applications, and fitness apps, have facilitated the rise of remote exercise guidance. Research supports the effectiveness of remote or home-based exercise programs as viable alternatives to traditional training methods.^[13–16]^ These digital programs offer several advantages, including reduced infection risks, flexible scheduling, and minimal space requirements.^[17]^ In this context, our study defines internet-based exercise as engaging in physical activity guided or supervised via online platforms, including websites and smartphone APPs.^[18,19]^ However, literature also suggests that team and outdoor exercises might offer enhanced mental health benefits, highlighting the limitations of virtual exercises in replicating the complete experience of outdoor activities and direct social interactions.^[20]^

Our research aims to address the gap in comprehensive studies examining the mental health benefits of online exercise interventions. Through a systematic review and meta-analysis, this study seeks to elucidate the potential advantages of Internet-driven exercise in mitigating depression and anxiety symptoms, hypothesizing that internet-based exercise can impact mental health. We also explore the variation in intervention effects across different populations, exercise durations, and methods.

## 2. Methods

This systematic review and meta-analysis is conducted under the guidance of the PRISMA 2020 statement.^[21]^

### 2.1. Eligibility criteria

The inclusion criteria for the articles are as follows: Only include those with randomized controlled trials. Full text published in English. Only include peer-reviewed journal articles. Intervention excludes any form of direct psychological guidance. Intervention is limited to exercise and physical activities. Studies that examined adults of 18 years and above without exercise disorder. Exercise interventions must be limited to web-based or smartphone APP-based. Anxiety and depression symptoms in the study could be clearly extracted.

### 2.2. Information sources and search strategy

Overall, 7 electronic databases were searched (Web of Science Core Collection, PubMed, PsycINFO, Medline, BIOSIS Previews, SPORTDiscus, and Education Source) to identify relevant studies published in English on September 1, 2022. Search terms include the following, either separately or in combination, “exercise,” “sport,” “physical activity” “mental health,” “depression,” “anxiety,” “mood,” in combination with “APP,” “Internet,” “web,” “software,” “remote,” “smartphone,” “mobile,” “e-coach,” and “online.” Table 1 gives an example of search strategies on PubMed. Reference of selected articles were also searched.

**Table 1:** Search strategy in PubMed.

Step	Search strategies
#1	“exercise” OR “PA” OR “sport” [Mesh]
#2	“exercise” OR “physical activity” OR “sport” [Text Word]
#3	#1 OR #2
#4	“mental health” OR “depression” OR “anxiety” OR “mood” [Mesh]
#5	“mental health” OR “depression” OR “anxiety” OR “mood” [Text Word]
#6	#4 OR #5
#7	“APP” OR “Internet ” OR “smart phone ” OR “software ” OR “e-coach” OR “mobile” OR “web” OR “remote” [Mesh]
#8	“application ” OR “Internet ” OR “smart phone ” OR “software ” OR “e-coach” OR “mobile” OR “web” OR “remote” [Text Word]
#9	#7 OR #8
#10	#3 AND #6 AND #9

### 2.3. Study selection

All search results were deduplicated by year, title, and author via Endnote X9.3.3. Following deduplicates, 2 authors (ZC and HH) independently screened the title and abstract of studies. After the preliminary screening, the 2 authors then reviewed all eligible articles for full text. Disagreements were resolved between the 2 authors. If there was no consensus, the corresponding author (ZYT) was contacted for a final decision. A PRISMA 2020 flow diagram of the search and selection process is presented.

### 2.4. Assessment of trial quality and data extraction

The quality of trials was assessed by 2 authors (ZC and HH)—to avoid bias of publications, we followed guidelines from the Cochrane Handbook for Systematic Reviews of Interventions,^[22]^ which has been proved to have good validity, inter-rater agreement and reliability. The data extraction of trials was carried out at the same time. The data of trials were collected in the following areas: Basic information of articles (author, date of publication, country, and study design). Information on the trial subjects (size of sample, age, sex, health condition). Intervention details (duration, types of exercises, approach of intervention). Outcomes (mean, SD or confidence interval [CI]). Measurement index (questionnaire types).

### 2.5. Synthesis and analysis

R software (version 4.2.2, “meta” package) was used to perform the meta-analysis. The estimation of heterogeneity was quantified as I^2^, with heterogeneity estimates of 25% (low heterogeneity), 50% (moderate heterogeneity), and 75% (high heterogeneity).^[23]^ The statistically significant differences between the research results were determined by Chi-square test. If *P* ≥ .1, I^2^ < 50%, the fixed-effect model was used for the meta-analysis, otherwise, e the random-effects model was used. Because of different measurement methods and ranges across all these studies, standardized mean difference (SMD) and a 95% CI were used for measuring the effect. Effect sizes were quantified as large SMD (>0.8), medium (0.5–0.8) or small (0.2 − 0.5).^[24]^

### 2.6. Subgroup analysis

Subgroup analyses were grouped based on the following factors: health condition, whether or not video guidance was included in intervention, approach of intervention, type of exercise, duration of exercise, and measurement index.

## 3. Results

### 3.1. Search strategy results

**Figure 1.**
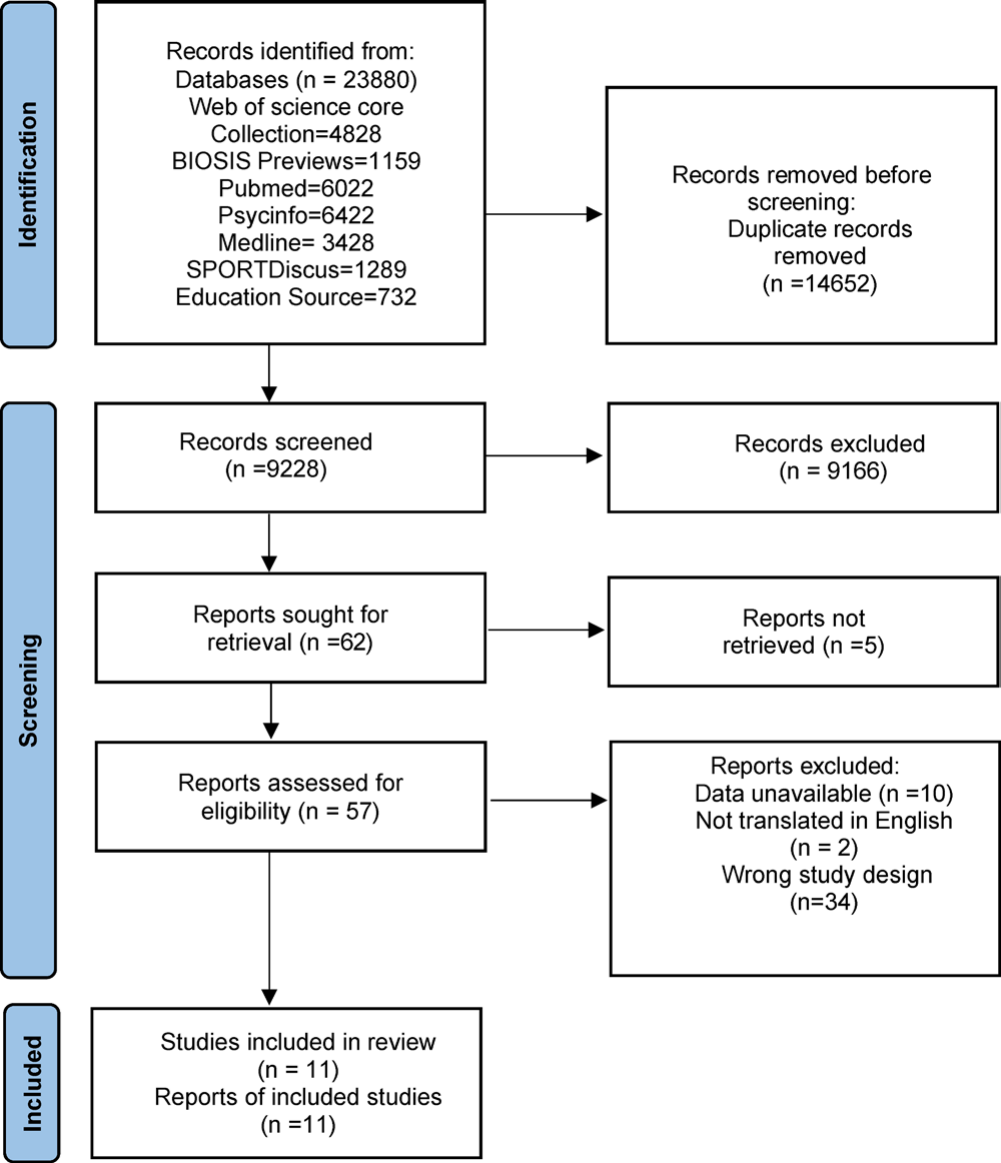
PRISMA 2020 flow diagram. The figure details the steps of search strategy and the selection of studies in this review. 11 articles were included for subsequent review.

The PRISMA 2020 flow diagram (Fig. 1) details the steps of search strategy and the selection of studies in this review. The initial search returned 23,880 articles on a database search. After deduplication, 9166 did not meet the criteria through reviewing the title and abstract. The remaining 57 articles were read in full text, 46 were eliminated mostly because they have no explicitly reported data on depression or anxiety measures, and finally 11 were included.

### 3.2. Study characteristics

General characteristics of the 11 studies are detailed in Table 2, including author and publication information, demographic information of the subjects, and the description of the exercise intervention.

**Table 2:** General characteristics.

No.	Yr	Author	Country	N	Female%	Age	Population characters	Measurement tool	Type of exercise	Approach of intervention	Duration (wk)
1	2013	Strom (35)	Sweden	48	83.3	48.8	People with depression	BDI	Physical activity promotion	Web	9
2	2014	Pilutti (36)	USA	78	24	48.4	People with multiple sclerosis	HADS	Physical activity promotion	Web	24
3	2016	Wahle (37)	Sweden	24	64	38.5	People with depression	PHQ-9	Physical activity promotion	Smart phone APP	8
4	2017	Nystrom (38)	Sweden	118	76	42	People with depression	PHQ-9	Physical activity promotion	Smart phone APP	12
5	2020	Edney (39)	Australia	270	75.2	41	Healthy adults	21-IDASS	Physical activity promotion	Smart phone APP	12 and 36
6	2020	Gur (40)	Turkey	128	87.5	20	Healthy adults	SF-36	Mix exercise	Smart phone APP	8
7	2021	Taylor (41)	UK	361	66	50	People with chronic health conditions	HADS	Physical activity promotion	Web	16 and 48
8	2021	Van (42)	Netherlands	92	50	59.3	Breast or prostate cancer survivors	POMS	Mix exercise	Web	24
9	2021	Wadhen (43)	UK	34	82	42.7	Healthy adults	DASS-21	Aerobic (Yoga)	Web	6
10	2022	Gao (44)	USA	40	100	56.9	Breast cancer survivors	5-PLS	Aerobic (Tai Chi)	Smart phone APP	12
11	2022	Magdalena (45)	Australia	84	71.4	44.2	People with Type 2 Diabetes	PHQ-9	Physical activity promotion	Smart phone APP	10 and 20

### 3.3. Quality of the evidence

**Figure 2.**
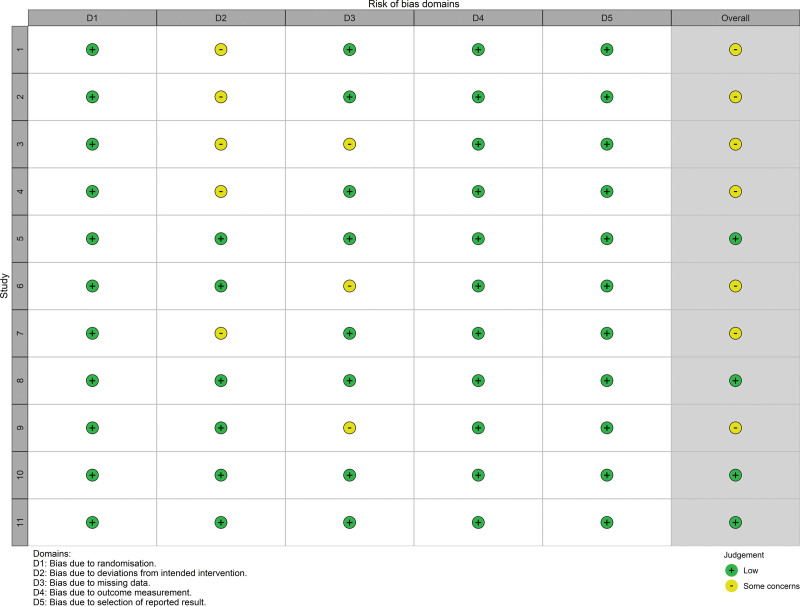
Risk of bias summary. Five studies were considered to have an ambiguous bias risk due to deviations from intended interventions, and 3 studies were found to have incomplete data.

**Figure 3.**
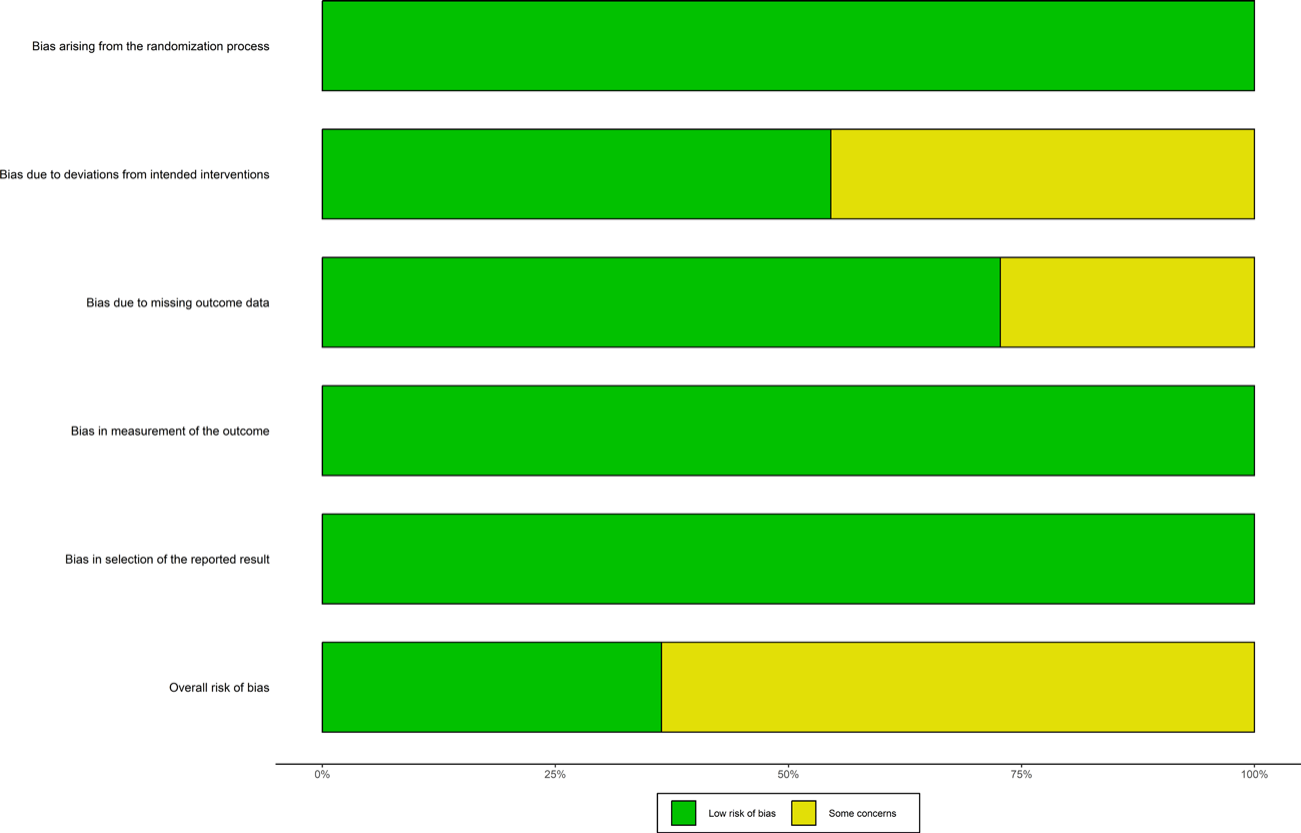
Risk of bias graph. Four studies have low risk, 7 studies have some concerns.

According to Version 2 of the Cochrane risk-of-bias tool for randomized trials (RoB 2),^[25]^ the quality of the evidence was assessed independently by 2 authors. For the controversial parts, the authors reached a consensus after discussion. After evaluating the risk of bias in the studies, 5 studies were considered to have an ambiguous bias risk due to deviations from intended interventions, and 3 studies were found to have incomplete data (Fig. 2). Overall, 4 studies have low risk, 7 studies have some concerns (Fig. 3).

### 3.4. Effects of internet-based exercise on depression and anxiety

**Figure 4.**
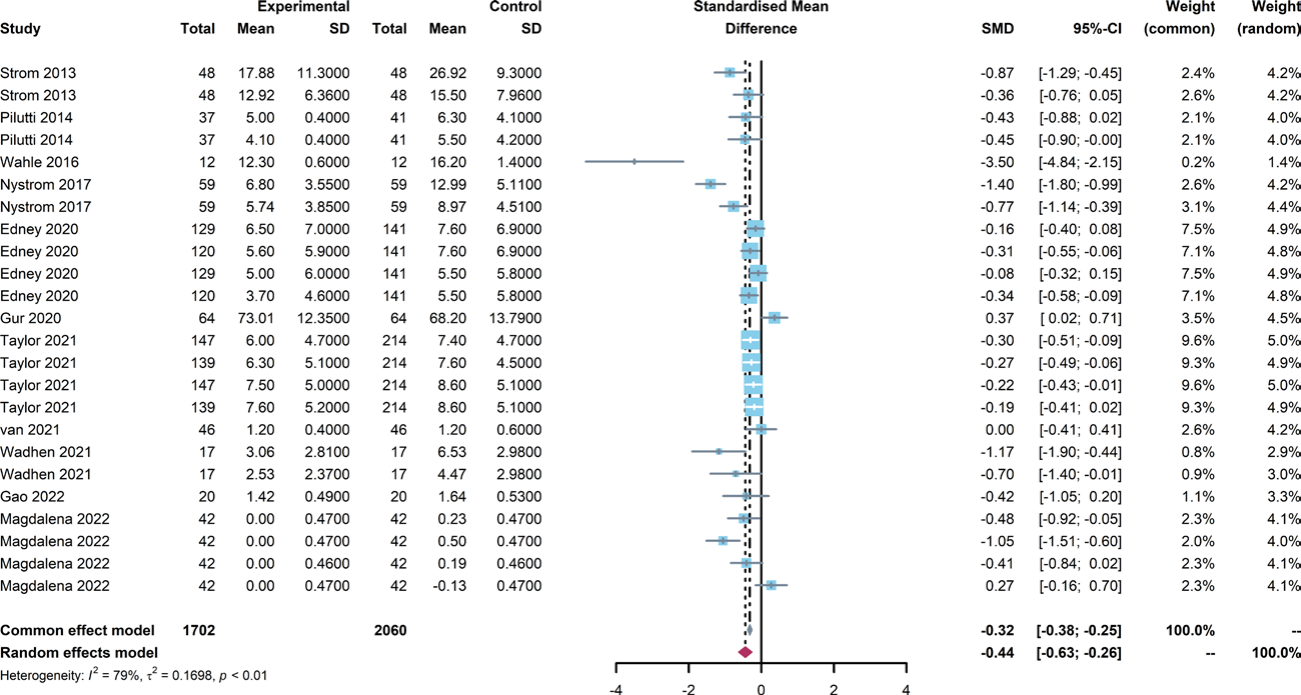
Forest plot of internet-based exercise effects on depression and anxiety. The overall effect of internet-based exercise: [SMD = −0.44, 95% CI (−0.63, −0.26), I^2^ = 79.3%, *P* < .01]. SMD = standardized mean differences.

According to meta-analysis (Fig. 4), compared to pre-intervention, people who were intervened with internet-based exercise showed a statistically significant reduction in depression and anxiety symptoms [SMD = −0.44, 95% CI (−0.63, −0.26), I^2^ = 79.3%, *P* < .01].

### 3.5. Subgroup analysis

Table 3 details the subgroup analysis assessing potential moderating factors for effects of internet-based exercise intervention.

**Table 3:** Subgroup analysis assessing potential moderating factors for effects of internet-290 based exercise intervention.

Population characteristics	Studies		Internet-based exercise vs control		
	No.	References	Pooled ES (95% CI)	I^2^ index (%)	*P*
Health condition					
Depression	7	Strom, 2013; Pilutti, 2014; Wahle, 2016; Nystrom, 2017	−0.96 (−1.55, −0.37)	82	<.01
Health	7	Edney, 2020; Gur, 2020; Wadhen, 2021	−0.26 (−0.54, 0.02)	72	<.01
Chronic	10	Taylor, 2021; Van, 2021; Gao, 2022; Magdalena, 2022	−0.29 (−0.46, −0.11)	58	<.05
Video					
Video included	7	Pilutti, 2014; Gur, 2020; Van, 2021; Gao, 2022; Wadhen, 2021	−0.34 (−0.70, 0.02)	82	<.01
No video	17	Strom, 2013; Wahle, 2016; Nystrom, 2017; Edney, 2020; Taylor, 2021; Magdalena, 2022	−0.48 (−0.71, −0.26)	73	<.01
**Approach of intervention**					
Web	11	Pilutti, 2014; Strom, 2013; Van, 2021; Taylor, 2021; Wadhen, 2021	−0.31 (−0.40,−0.22)	45	<.05
Smartphone APP	13	Gur, 2020; Gao, 2022; Wahle, 2016; Nystrom, 2017; Edney, 2020; Magdalena, 2022	−0.52 (−0.90,−0.14)	87	<.01
**Type of exercise**					
Physical activity promotion	19	Strom, 2013; Pilutti, 2014; Wahle, 2016; Edney, 2020; Nystrom, 2017; Taylor, 2021; Magdalena, 2022	−0.47 (−0.66,−0.27)	79	<.05
Aerobic	3	Gao, 2022; Wadhen, 2021	−0.73 (−1.15,−0.31)	13	.32
Mix	2	Gur, 2020; Van, 2021	0.20 (−0.16, 0.56)	44	.18
**Duration**					
<12 wk	8	Strom, 2013; Wahle, 2016; Magdalena, 2022; Gur, 2020; Wadhen, 2021	−0.76 (−1.38, −0.14)	86	<.01
12−24 wk	12	Pilutti, 2014; Nystrom, 2017; Van, 2021; Edney, 2020; Gao, 2022; Magdalena, 2022	−0.40,(−0.66, −0.15)	82	<.01
>24 wk	4	Taylor, 2021; Edney, 2020	−0.27 (−0.39, −0.16)	0	.83
**measurement index**					
Depression	12	Strom, 2013; Pilutti, 2014; Wahle, 2016; Nystrom, 2017; Edney, 2020; Wadhen, 2021; Taylor, 2021; Van, 2021; Magdalena, 2022	−0.67 (−1.04, −0.30)	86	<.01
Anxiety	10	Strom, 2013; Pilutti, 2014; Nystrom, 2017; Edney, 2020; Wadhen, 2021; Taylor, 2021; Magdalena, 2022	−0.29 (−0.39, −0.20)	45	<.01
Depression and anxiety	2	Gur, 2020; Gao, 2022;	0.02 (−0.75, 0.87)	78	<.05

CI = confidence interval, ES = effect size, I^2^ = heterogeneity, No. = number of the included studies, *P* = test for overall effect.

#### 3.5.1. Subgroup: Health condition.

Considering the potential influence of participants’ own health conditions on their psychological states, this study categorized the sample into 3 groups based on their health status. The mental disorder group refers to participants who reported having diagnosed mental disorders (such as depression). The physical disorder group includes participants who reported having diagnosed chronic organic physical disorders, primarily encompassing conditions like cancer, diabetes, multiple sclerosis, etc. The healthy adult group refers to participants who did not report any form of physical or mental disorders in the study. It can be observed that people with depression [SMD = −0.96, 95% CI (−1.55, −0.37), I^2^ = 82%, *P* < .01] have statistically significant improved through internet-based exercise. Similarly, there was statistical significance in other groups, but the SMD of the physical disorder group [SMD = −0.29, 95% CI (−0.46, −0.11), I^2^ = 58%, *P* = .01] and the healthy adult group [SMD = −0.26, 95% CI (−0.54, 0.02), I^2^ = 72%, *P* < .01] were lower than people in the mental disorder group. There was no statistically significant subgroup effect (*P* = .09).

#### 3.5.2. Subgroup: Whether video guidance is included in intervention.

Studies were divided into 2 groups based on whether video guidance is included in intervention. It can be observed that the video group [SMD = −0.34, 95% CI (−0.70, 0.02), I^2^ = 82%, *P* < .01] and non-video group [SMD = −0.48, 95% CI (−0.71, −0.26), I2 = 73%, *P* < .01] were statistically significant through the subgroup analysis. There was no statistically significant subgroup effect (*P* = .43).

#### 3.5.3. Subgroup: Approach of intervention.

Based on the approach of intervention, studies were divided into 2 groups. The web group included any form of intervention with PC as the terminal (such as email, website, web video, etc.). The smart phone APP group included those guided by exercise intervention in the form of mobile phone APPs. It can be observed that the web group [SMD = −0.31, 95% CI (−0.40,−0.22), I^2^ = 45%, *P* = .05] and the smart phone APP group [SMD = −0.52, 95% CI (−0.90,−0.14), I^2^ = 87%, *P* < .01] were statistically significant through the subgroup analysis. Although the SMD of the smart phone APP group was slightly higher than the web group, there was no statistically significant subgroup effect (*P* = .35).

#### 3.5.4. Subgroup: Type of exercise.

Based on the type of exercise, studies were divided into 3 groups. It can be observed that physical activity promotion [SMD = −0.47, 95% CI (−0.66,−0.27), I^2^ = 79%, *P* < .01] was statistically significant through subgroup analysis, aerobic [SMD = −0.73, 95% CI (−1.15,−0.31), I^2^ = 13%, *P* = .32] and mix exercise [SMD = 0.20, 95% CI (−0.16, 0.56), I2 = 44%, *P* = .18] were not statistically significant. There was a statistically significant subgroup effect (*P* < .01).

#### 3.5.5. Subgroup: Duration.

Based on the duration of the exercise intervention, studies were divided into 3 groups, with the duration range of < 12 weeks, 12 to 24 weeks, and > 24 weeks, respectively. It can be observed that < 12 weeks [SMD = −0.76, 95% CI (−1.38,−0.14), I^2^ = 86%, *P* < .01] and 12 to 24 weeks [SMD = −0.40, 95% CI (−0.66,−0.15), I^2^ = 82%, *P* < .01] were statistically significant through subgroup analysis. There was no statistical significance in the > 12 weeks group [SMD = −0.27, 95% CI (−0.39,−0.16), I^2^ = 0%, *P* = .83]. There was a statistically significant subgroup effect (*P* = .01).

#### 3.5.6. Subgroup: Measurement index.

According to the measurement index, studies were divided into 3 groups, including the depression group, the anxiety group, and the depression and anxiety group. The depression and anxiety group was defined by the comprehensive mental health indicators measured in the trail, including depression and anxiety. It can be observed that the depression group [SMD = −0.67, 95% CI (−1.04,−0.30), I^2^ = 86%, *P* < .01], the anxiety group [SMD = −0.29, 95% CI (−0.39,−0.20), I^2^ = 45%, *P* = .01], and the depression and anxiety group [SMD = 0.02, 95% CI (−0.75, 0.87), I^2^ = 78%, *P* = .03] were statistically significant through subgroup analysis. There was no statistically significant subgroup effect (*P* = .13).

### 3.6. Publication bias

**Figure 5.**
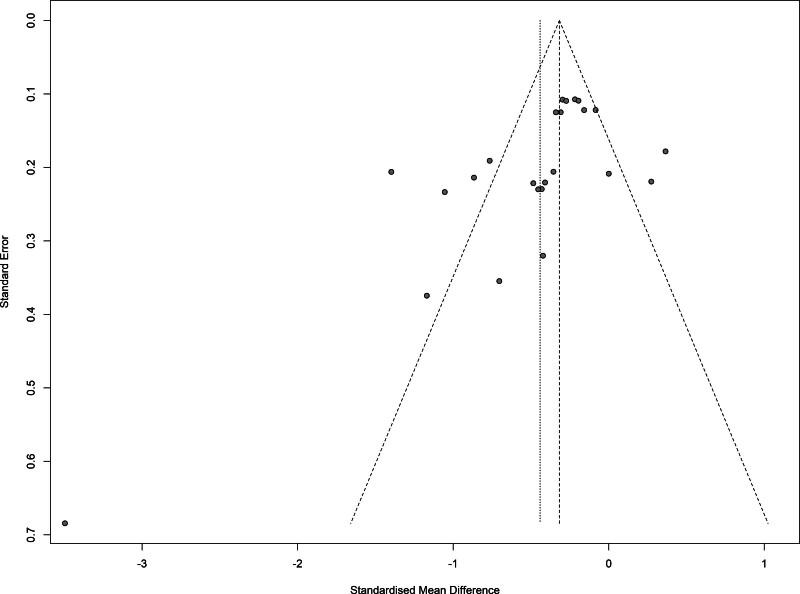
Funnel plot of publishing bias. The funnel plot appears asymmetric.

The possible publication bias is indicated by the funnel plot (Fig. 5). Since correlations between continuous type outcome and both effect size and its standard error exist, the funnel plot can appear highly asymmetric, even when no publication bias is present.^[26]^ The result of the Egger test (t = −2.91, *P* < .01) shows the publication bias of studies was statistically significant.

A sensitivity analysis (leave-one-out meta-analysis) was also conducted to test the stability of studies. After sequentially removing each study, no studies affecting heterogeneity were found (I^2^ = 73%–80%). The stability of the results was confirmed.

## 4. Discussion

This meta-analysis seeks to determine the effects of internet-based exercise on depression and anxiety. Incorporating data from 11 studies and a total of 1009 participants, our findings consistently indicate that internet-based exercise interventions produce statistically significant ameliorations in symptoms of depression and anxiety, with an overall SMD of −0.44 [95% CI (−0.63, −0.26), I^2^ = 79.3%, *P* < .01]. These results contribute to a growing body of literature that emphasizes the mental benefits of internet-based exercise across various populations, and are congruent with earlier meta-analytic findings which have delineated the impacts of physical activity on depression (SMD = −0.50) and anxiety (SMD = −0.38).^[12]^

Interestingly, our data show pronounced benefits of internet-based exercise interventions among depressed patients, with an effect size of SMD = −0.96. This is notably greater than that observed in healthy individuals (SMD = −0.29) and those with chronic diseases (SMD = −0.26). Such variations in effect sizes can likely be attributed to floor effects,^[27]^ suggesting that individuals with more pronounced psychological distress have greater potential for improvement. Multiple studies have endorsed the therapeutic potential of exercise for depressed individuals.^[28,29]^ This study corroborates that notion by suggesting internet-based exercise can serve as a potent alternative for depression and anxiety relief.

The exercise interventions examined predominantly comprised prescribed exercise forms—ranging from guidelines on physical activity, stipulated walking durations, to specific modalities like Yoga and Tai Chi. It was observed that structured aerobic exercises are more efficacious in mitigating depression and anxiety compared to mere physical activity promotion, with exercise intensity potentially explaining this disparity. Both Henriksson^[30]^ and Ji^[31]^ have postulated that while both low and high-intensity exercises can enhance psychological well-being, the benefits are more pronounced with high-intensity regimes. In this study, all 3 aerobic exercises included achieved good psychological benefits (SMD = −0.73), but due to the relatively small number of studies, the SMD of aerobic exercise was not statistically significant (*P* > .05). This benefit may come from the intensity of exercise, moderate-intensity exercise may be an optimal intensity of exercise for the promotion of mental health.^[32]^ Interesting types of exercise may also increase the psychological benefits of exercise. For mixed exercise, although research has confirmed the psychological benefits of resistance training,^[33]^ the sample of one trail is cancer survivors who may be affected by their health condition and have significant psychological pressure. The intervention method of the other trial is mainly resistance training, so there may be significant publication bias.

Another pivotal facet of this discussion is the exercise duration. Our data suggest that the mental health dividends of exercise wane over time, with internet-based interventions demonstrating the most profound impact when limited to <12 weeks (SMD = −0.76). Stanton meta-analysis echoes this, emphasizing the efficacy of interventions spanning 4 to twelve weeks in attenuating depressive symptoms.^[34]^ Conversely, Blumenthal posits that though antidepressant medications manifest rapid initial effects, exercise parallels their efficacy post 16 weeks of treatment.^[35]^ In this study, the impact of prolonged exercise intervention on mental health was relatively insignificant (SMD = −0.27, *P* > .05). Wang meta-analysis also reached a similar conclusion that exercise interventions over 16 weeks did not have relatively significant psychological benefits.^[36]^ Therefore, we speculate that there is a certain dose-response relationship between exercise intervention and psychological benefits, and the psychological benefits of exercise may be adaptive, and after developing exercise habits, it may be necessary to change the intensity or type of exercise to generate more psychological stimulation, but currently there are relatively few studies on long-term exercise intervention, so the specific mechanism is not yet clear. In addition, these disparities could stem from variations in exercise intensity and the target demographics. A holistic understanding of the exercise prescription in mental health contexts demands further exploration, especially considering variables like intensity, duration, and frequency. Our findings, when juxtaposed with prior studies, suggest that the mental health effect SMD of internet-based exercise is somewhat lesser than other forms, including aquatic exercise (SMD = −0.77),^[37]^ aerobic exercise (SMD = −0.53),^[38]^ and Yoga.^[39]^

This study is not without limitations. The remote nature of interventions meant that control over exercise intensity and type was challenging. Additionally, our sample predominantly comprised females, which could induce gender-related biases. The inclusion of non-randomized controlled trials also introduces the potential for selection bias. Acknowledging these constraints, we advocate for more expansive, rigorous research in the forthcoming years.

## 5. Conclusion

The effects of internet-based exercise on mental health are promising, demonstrating a statistically significant improvement. In particular, internet-based exercise interventions appear to be especially beneficial as an adjunctive treatment for individuals with depression. Shorter durations, specifically those under 12 weeks of internet-based aerobic exercise, seem to have a more pronounced positive impact on symptoms of depression. Nevertheless, the current conclusions are based on a limited dataset. For robust validation of these findings, future research should involve larger sample sizes and higher-quality studies.

## Author contributions

**Conceptualization:** Zuo Chen, Zhengyan Tang.

**Data curation:** Zuo Chen, Hui Huang, Ruidong Liu, Zhengyan Tang.

**Methodology:** Zuo Chen, Zhengyan Tang.

**Software:** Hui Huang, Ruidong Liu.

**Writing –original draft:** Zuo Chen, Hui Huang, Ruidong Liu, Zhengyan Tang.

**Writing – review & editing:** Zuo Chen, Hui Huang, Ruidong Liu, Zhengyan Tang.
